# A 2-year field trial reveals no significant effects of GM high-methionine soybean on the rhizosphere bacterial communities

**DOI:** 10.1007/s11274-018-2495-7

**Published:** 2018-07-09

**Authors:** Jingang Liang, Yue Jiao, Ying Luan, Shi Sun, Cunxiang Wu, Haiying Wu, Mingrong Zhang, Haifeng Zhang, Xiaobo Zheng, Zhengguang Zhang

**Affiliations:** 1 Department of Plant Pathology, College of Plant Protection, Nanjing Agricultural University, and Key Laboratory of Integrated Management of Crop Diseases and Pests, Ministry of Education, Nanjing, People’s Republic of China; 2 Development Center of Science and Technology, Ministry of Agriculture and Rural Affairs, Beijing, People’s Republic of China; 3 The National Key Facility for Crop Gene Resources and Genetic Improvement (NFCRI), MOA Key Laboratory of Soybean Biology (Beijing), Institute of Crop Science, The Chinese Academy of Agricultural Sciences, Beijing, People’s Republic of China; 4 Nanchong Academy of Agricultural Science, Nanchong, People’s Republic of China

**Keywords:** GM soybean, Methionine content, Bacterial population, 16S rRNA gene sequencing

## Abstract

Genetically modified (GM) crops have brought various economic benefits but may also have adversely affected soil microorganisms. To examine whether transgenic high-methionine soybean ZD91 alters the bacterial community structure in the rhizosphere, we performed a 2-year follow-up study using the transgenic high-methionine soybean cultivar ZD91 and wild type cultivar ZD. The community composition and the relative abundance of bacteria in rhizosphere soil were determined by sequencing of the 16S rRNA amplicon. Our results indicated that transgenic soybean ZD91 had no significantly effects on rhizosphere bacterial communities. Instead, the plant growth stage and year appeared to have a stronger effect on bacterial communities. Our findings therefore provided reliable scientific evidence for potential commercial cultivation of cultivar ZD91.

## Introduction

Soybean [*Glycine max* (L.) Merr.] has high protein contents and is often used as the superior plant origin protein source (Grazina et al. 2017). However, its low contents of sulfur-containing amino acid limit its nutrition values that results in efforts to potentially increase the levels of methionine in soybean (Amir et al. 2012; Song et al. 2013). Recently, a transgenic high-methionine soybean cultivar ZD91 has been produced that boosts methionine contents by 2.3-fold in dried soybeans (Song et al. 2013). The cultivation of the transgenic high-methionine soybean raises the concerns over its safety, as it is generally applied to genetically modified (GM) crops in general (Hilbeck et al. 2015). It is important to evaluate the impact of GM crops for human safety and the environment, including the potential impact on soil ecosystems prior to commercial cultivation (Kostov et al. 2014; Liang et al. 2014a; Li et al. 2014; Liu et al. 2005; Song et al. 2014; Vilvert et al. 2014).

Rhizosphere bacteria play an important role in the development of plants, which are often influenced by plant genotypes and environmental conditions (Filion 2008). Changes in microbial community diversity may influence bio- and geochemical processes and soil ecology (Chauhan et al. 2014; Kostov et al. 2014). Crops interact with soil microorganisms, and thus affect microbial community dynamics within the rhizosphere (Wu et al. 2014). It is also suggested that rhizosphere microbial communities are more sensitive to GM crops than those in bulk soil (Shen 2015). For these reasons, the bacterial community structure in the rhizosphere soil is often used as an early and sensitive indicator for assessing the effects of GM crops on soil ecology. Previous studies have revealed little effects of genetically engineered plants on rhizosphere microbial communities. Weinert et al. (2009) showed that the effects of GM potato plants were negligible when compared to the effects of environmental factors, including the field site or growth year. Hur et al. (2011) found that the effect of GM poplar on microbial indicators was not biologically significant. Mansouri et al. (2002) demonstrated that the induction of the opine-utilizing community in the rhizosphere was independent of the GM plant species. However, contradictory finding also existed as Di Giovanni et al. (1999) found a difference in carbon substrate utilization patterns between rhizosphere bacterial communities of the parental and lignin peroxidase transgenic alfalfa. These studies also indicated that evaluating the environmental risks of GM crops is dependent on a case-by-case basis (Andow and Hilbeck 2004).

Our previous study showed that high-methionine soybean cultivar ZD91 has no significant impact on bacterial communities of the rhizosphere at a single plant growth stage (Liang et al. 2014b), but the long-term effect remains unclear. It is advised that the safety assessment of GM crops should be conducted over at least 2 years, and more comprehensive assessments of risks over longer temporal and larger spatial scales are recomended (Filion 2008; Woodbury et al. 2017). This assesses the environmental impact of transgenic soybean ZD91 over a 2-year period to provide the evidence supporting the commercial cultivation.

## Materials and methods

### Plant materials

The high-methionine soybean cultivar ZD91 was developed by introducing an *Arabidopsis thaliana* cystathionine γ-synthase gene into soybean cultivar Zigongdongdou (ZD) using *Agrobacterium*-mediated transformation. The parental ZD was used as a control. There were no significant differences in other agronomic traits between transgenic (ZD91) and wild type (ZD) (Song et al. 2013).

### Field design, rhizosphere sampling, and soil DNA extraction

Field trial experiments were performed over two consecutive years (2012–2013, i.e., the 3rd and 4th year of the experiment) at Nanchong (30°48′N, 106°04′E), Sichuan, China (Liang et al. 2016). Four replicated plots for each cultivar (ZD and ZD91) were established in a randomized design. Sampling was carried out at various stages of the growth cycle, corresponding to the seedling, flowering, pod-setting, and maturity-setting stages each year. Rhizosphere samples were collected according to previous report (Liang et al. 2015).

DNA was extracted from 0.5 g of rhizosphere soil samples using the MoBio PowerSoil DNA Isolation Kit (MoBio Laboratories, Inc., USA). The final quantity and quality of DNA was evaluated using a NanoDrop 1000 Spectrophotometer (Thermo Scientific, USA) (Liang et al. 2015). Extracted DNA of high-quality (OD260/280 = 1.8–2.0, c ≥ 20 ng/µL) was adjusted to a concentration of 20 ng/µL and used for PCR amplification.

### Sequencing library construction

Bacterial 16S rRNA gene amplification and sequencing were performed at BGI (Shenzhen, China). In brief, amplification of the 16S V4 region was accomplished using a dual index paired-end sequencing strategy in an Illumina MiSeq platform as described by Kozich et al. (2013). Each primer consisting of an Illumina adapter, an 8-nt index sequence, 10-nt pad sequences, a 2-nt linker, and gene-specific primers (515F 5′-GTGCCAGCMGCCGCGGTAA-3′ and 806R 5′-GGACTACHVGGGTWTCTAAT-3′). Amplification was performed on a 96-well plate using AccuPrime Pfx SuperMix reagents, and library clean-up and normalization were performed using the Invitrogen SequalPrep Plate Normalization Kit. The library QC was performed using a KAPA Biosystems Q-PCR kit and by obtaining a bioanalyzer trace using the Agilent Technologies HS DNA kit (Ryan et al. 2016).

### Data processing and analysis

Paired-end reads of FASTQ files are available in the SRA under BioProject PRJNA284221. Sequences were analyzed using Mothur v1.31.2 (Schloss et al. 2009). Briefly, all reads were aligned with reference database SILVA alignment (v102) using the NAST algorithm, and assigned to 16S V4 region (Pruesse et al. 2007). Chimeras were removed using UCHIME v4.2 (Edgar et al. 2011). For phylotype identification, sequences were taxonomically classified at an 80% confidence to the Mothur-adapted RDP database (v9) using the previous described method (Wang et al. 2007). The sequences that either were not classified into the level of kingdom or that classified as *Archaea, Eukaryota*, chloroplasts, or mitochondria were culled (Jünemann et al. 2012). Operational taxonomic units (OTUs) were identified using a 97% similarity rate and used for downstream community analyses. To avoid species overestimation, all singleton OTUs were excluded (Jünemann et al. 2012). The bacterial OTUs that occurred in more than 48 samples (frequency > 75%) were defined as the common OTUs (Liang et al. 2014b).

### Statistical analysis

One-way ANOVA and Duncan pair-wise comparisons (*P* < 0.01) were used to determine the minimum significant difference by employing SPSS 17.0 software (Liang et al. 2015). The richness estimators (ACE and Chao), diversity indices (Shannon and Simpson), and rarefaction curve were generated using Mothur v1.31.2. Principal component analysis (PCA) was performed in R to compare bacterial community structure across all samples. Variation in OTU composition between cultivar, growth stage and year, and the interactions among them were tested for significance using the ADONIS function in vegan. Variance partitioning analysis (VPA) was used to determine the contributions of cultivar, growth stage and year, as well as interactions among them on the variations in a bacterial community structure with Hellinger-transformed data.

## Results

### Illumina MiSeq sequence analysis of the rhizosphere bacterial communities

**Fig. 1:**
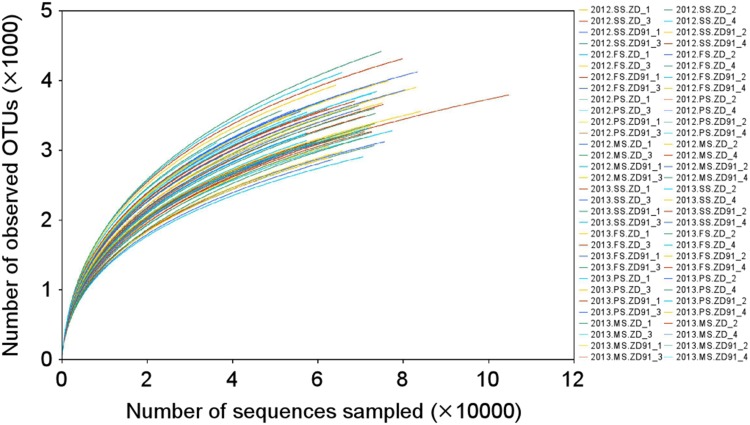
Rarefaction analysis. The rarefaction curve of the OTUs obtained from the soybean rhizosphere soil of various cultivars. The curves were named in the following form: “year. growth stage. cultivar replicate”. *SS* seedling stage, *FS* flowering stage, *PS* pod-setting stage, and *MS* maturity-setting stage

To analyze the composition of the rhizosphere bacterial communities, we sequenced 16S rRNA amplicons. In total, 3,920,326 high-quality sequences were obtained, with an average of 61,255 sequences per sample. The cultivars were grouped based on the number of replicates (ZD_1, ZD_2, ZD_3, and ZD_4 for ZD, and ZD91_1, ZD91_2, ZD91_3, and ZD91_4 for ZD91). The rarefaction curves showed no differences between ZD and ZD91 in both years, and the data were sufficient for revealing differences, if any, between the cultivars (Fig. 1). There were also no significant differences in the estimators of community richness (observed species, Chao, and ACE) and diversity (Shannon and Simpson) between ZD and ZD91 (Table 1).

**Table 1:** Sequencing data summary (means ± standard errors) (*P* < 0.01)

	Seedling stage (SS)		Flowering stage (FS)		Pod-setting stage (PS)		Maturity-setting stage (MS)	
	ZD	ZD91	ZD	ZD91	ZD	ZD91	ZD	ZD91
2012								
OTUs	3173.25 ± 95.24	3362.75 ± 174.17	3100.50 ± 125.47	2795.75 ± 159.57	3294.50 ± 323.83	3186.75 ± 163.90	3348.75 ± 216.84	3214.25 ± 257.94
Chao	4476.04 ± 163.75	4713.92 ± 240.00	4493.24 ± 132.43	4039.74 ± 251.83	4750.65 ± 352.50	4565.96 ± 93.41	4887.93 ± 324.14	4488.93 ± 253.62
ACE	4892.25 ± 476.14	5014.09 ± 569.10	5039.16 ± 248.09	4650.81 ± 437.91	5461.41 ± 130.86	4968.38 ± 442.59	5318.38 ± 783.44	4933.32 ± 331.11
Shannon	6.07 ± 0.08	6.09 ± 0.09	5.92 ± 0.09	5.80 ± 0.07	5.96 ± 0.14	5.92 ± 0.07	6.07 ± 0.09	6.05 ± 0.08
Simpson	0.01 ± 0.00	0.01 ± 0.00	0.01 ± 0.00	0.01 ± 0.00	0.01 ± 0.00	0.01 ± 0.00	0.01 ± 0.00	0.01 ± 0.00
2013								
OTUs	3654.75 ± 132.23	3421.25 ± 517.78	3254.25 ± 535.29	3394.25 ± 594.15	3671.25 ± 535.81	3648.25 ± 241.77	3947.75 ± 321.54	3553.75 ± 729.51
Chao	5165.12 ± 159.56	4830.44 ± 581.42	4670.33 ± 624.22	4841.82 ± 738.93	5098.32 ± 566.25	5139.86 ± 342.49	5471.30 ± 353.77	5204.04 ± 796.83
ACE	5241.76 ± 136.72	5292.30 ± 540.93	5304.66 ± 608.72	5334.55 ± 607.29	5174.01 ± 642.27	5358.42 ± 421.66	5522.99 ± 354.98	5492.14 ± 725.90
Shannon	6.34 ± 0.06	6.27 ± 0.07	6.15 ± 0.08	6.09 ± 0.10	6.30 ± 0.03	6.31 ± 0.06	6.46 ± 0.08	6.47 ± 0.06
Simpson	0.01 ± 0.00	0.01 ± 0.00	0.01 ± 0.00	0.01 ± 0.00	0.01 ± 0.00	0.01 ± 0.00	0.01 ± 0.00	0.01 ± 0.00

The number of OTUs (observed species), richness estimators Chao and ACE, diversity estimators Shannon and Simpson were calculated at 3% distance

### Taxonomic composition

**Fig. 2:**
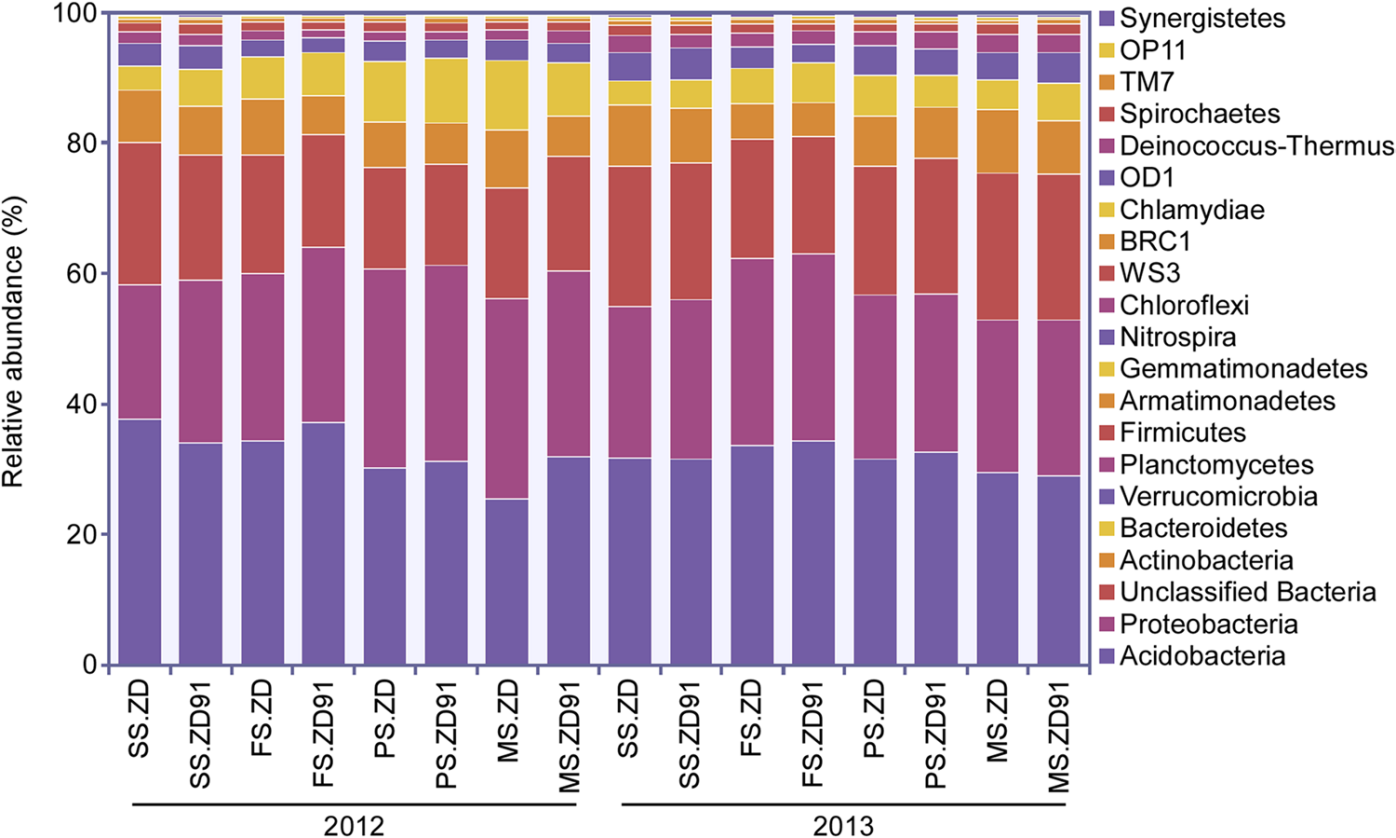
Bacterial composition at the phylum level. Relative read abundance of bacterial phyla within the communities. *SS* seedling stage, *FS* flowering stage, *PS* pod-setting stage, and *MS* maturity-setting stage

All sequences were classified according to the RDP classifier using the default settings. Twenty phyla were identified in the samples; the overall bacterial community structure at the phylum level for ZD and ZD91 was shown in Fig. 2. Acidobacteria, Proteobacteria, Actinobacteria, Bacteroidetes, Verrucomicrobia, Planctomycetes, and Firmicutes accounted for > 79% of the reads, but no significant difference was detected between ZD and ZD91 in the proportions of these phyla (Fig. 2). The percentage of unclassified bacteria was 19%.

3,920,326 bacterial sequences were then grouped into 13,178 OTUs at a 97% similarity threshold. A total number of 1831 common bacterial OTUs was detected in the rhizosphere soil. Among the common OTUs, 412 belonged to Proteobacteria, 200 to Acidobacteria, 165 to Actinobacteria, 131 to Planctomycetes, 97 to Bacteroidetes, 73 to Verrucomicrobia, 42 to Firmicutes, 34 to Armatimonadetes, 28 to Gemmatimonadetes, 19 to Chloroflexi, 5 to Nitrospira, 3 to BRC1, and 1 to WS3. These OTUs accounted for 94.75% sequences (3,714,645 out of 3,920,326).

**Fig. 3:**
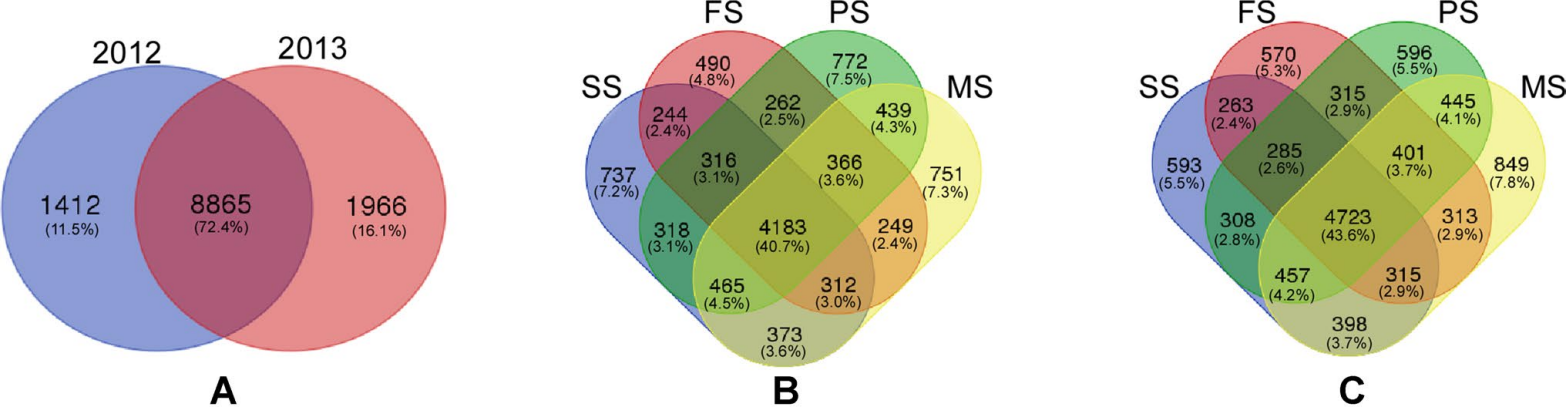
Venn diagram. **a** Venn diagram showing variable overlaps between the year 2012 and 2013. **b** Venn diagram showing variable overlaps between four growth stages in year 2012. **c** Venn diagram showing variable overlaps between four growth stages in year 2013. The numbers within the circles represent the OTUs numbers. *SS* seedling stage, *FS* flowering stage, *PS* pod-setting stage, and *MS* maturity-setting stage

Interestingly, 8865 (72.4%) OTUs were observed between year 2012 and year 2013. 1412 (11.5%) OTUs existed only in year 2012, and 1966 (16.1%) OTUs existed only in year 2013 (Fig. 3a).

Of the 10,277 OTUs identified from year 2012, 4183 (40.7%) OTUs occurred in all four different growth stages, 737 (7.2%) OTUs existed only in the seedling stage, 490 (4.8%) OTUs existed only in the flowering stage, 772 (7.5%) OTUs existed only in the pod-setting stage, and 751 (7.3%) OTUs existed only in the maturity-setting stage (Fig. 3b).

Of the 10,831 OTUs present in year 2013, 4723 (43.6%) existed in all four different growth stages, 593 (5.5%) OTUs were identified in the seedling stage, 570 (5.3%) OTUs were identified in the flowering stage, 596 (5.5%) OTUs were identified in the pod-setting stage, and 849 (7.8%) OTUs were identified in the maturity-setting stage (Fig. 3c). Proteobacteria were the most dominant phyla regardless of the year or the plant stage.

### The core genera

**Fig. 4:**
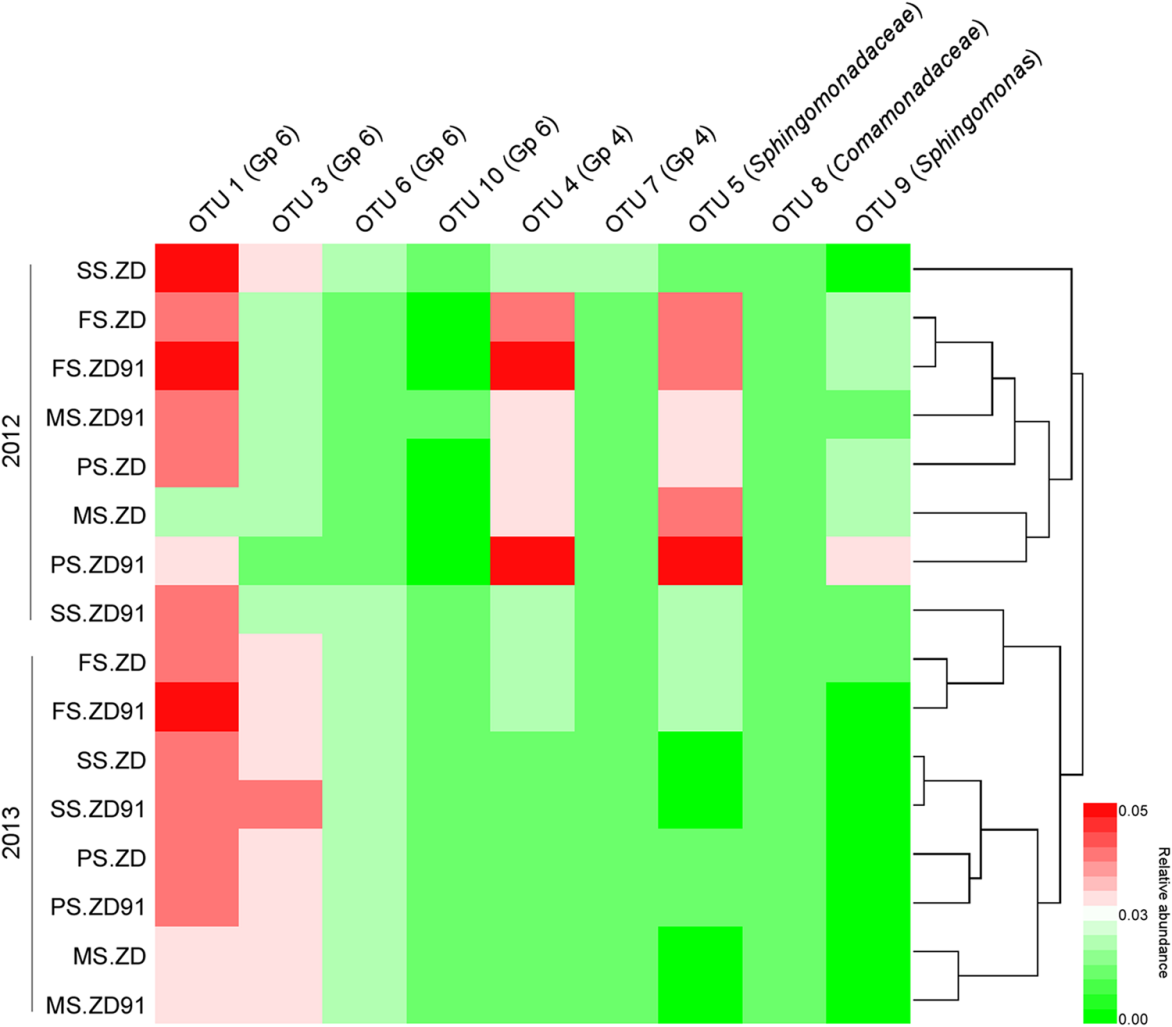
Heatmap of the most characteristic taxa (i.e. dominant core genera). Red and green indicate higher and lower frequency, respectively. *SS* seedling stage, *FS* flowering stage, *PS* pod-setting stage, and *MS* maturity-setting stage

To gain an insight into the impact of cultivation year and growth stage on bacterial communities, we applied heatmap analysis for 9 core OTUs (relative abundance > 1%), which highlights relative distribution and abundance (Fig. 4) (Guan et al. 2016). The results showed that cultivation year has a significant effect on the most characteristic taxa, except for OTU 1 (Gp6), OTU 10 (Gp6), OTU 7 (Gp4), and OTU 8 (*Comamonadaceae*). The effect of growth stage on the most characteristic taxa was most significant, except for OTU 7 (Gp4) in year 2012, and OTU 3 (Gp6), OTU 6 (Gp6), and OTU 7 (Gp4) in year 2013. There were no significant differences found in core genera during the same growth stage between ZD and ZD91.

OTU 1 (Gp6), OTU 4 (Gp4), OTU 5 (*Sphingomonadaceae*) were the most dominant core genera in year 2012, while OTU 1 (Gp6) and OTU 3 (Gp6) were the dominant core genera in year 2013. In the seedling stage, OTU 1 (Gp6) and OTU 3 (Gp6) were the dominant core genera. OTU 1 (Gp6), OTU 3 (Gp6), OTU 4 (Gp4), OTU 5 (*Sphingomonadaceae*) were the dominant core genera in other three stages.

### Bacterial community dynamics

**Fig. 5:**
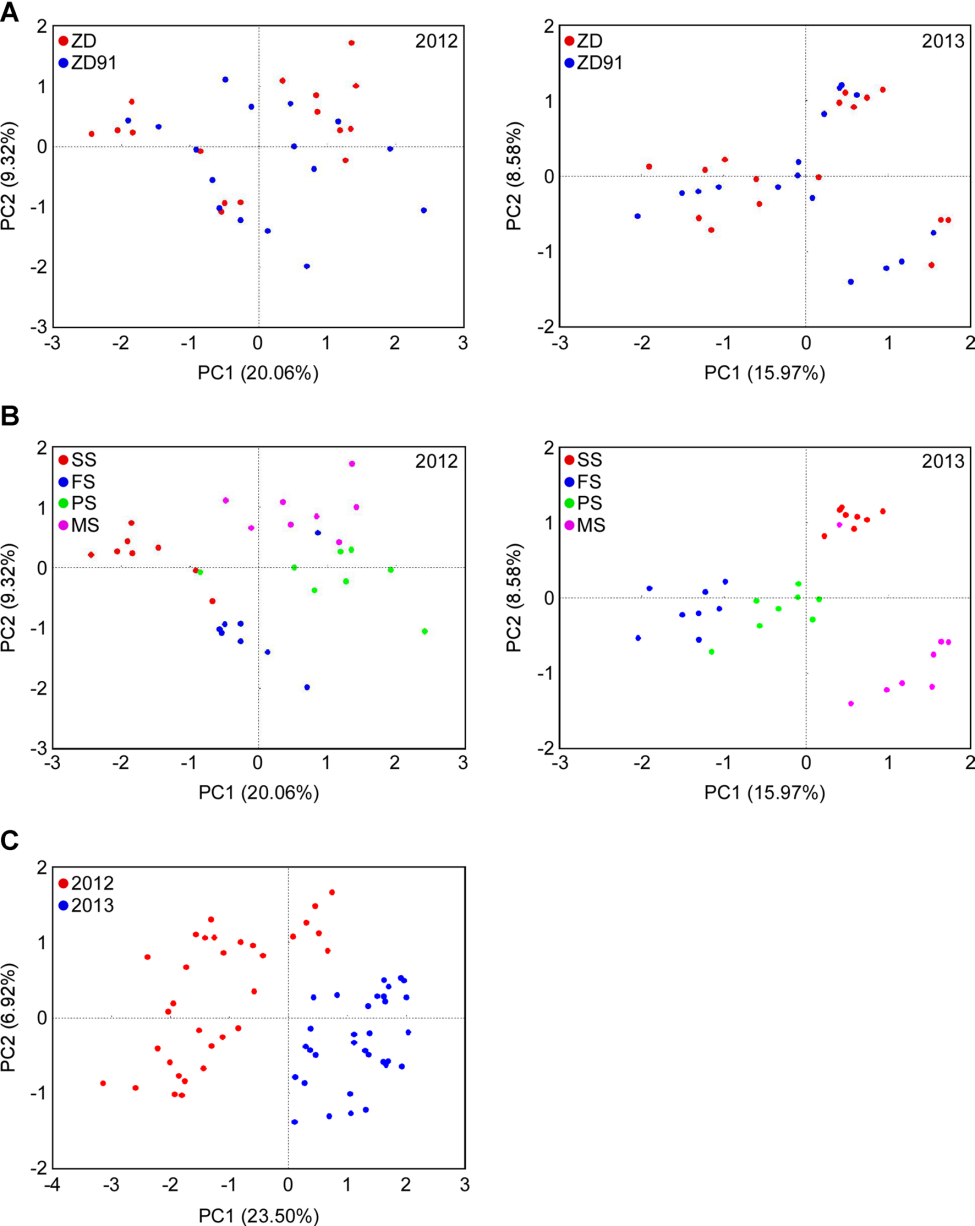
PCA of bacterial community structure based on OTUs at a distance of 3% for individual samples. **a** Bacterial community structure between cultivars. **b** Bacterial community structure of different growth stages. **c** Bacterial community structure between various growth years. The eigenvalues displayed on the diagram axes refer to the percentage variation of the respective axis. *SS* seedling stage, *FS* flowering stage, *PS* pod-setting stage, and *MS* maturity-setting stage

PCA was performed to compare the bacterial community differences between cultivars, growth stages, and growth years (Fig. 5). Using cultivar as an explanatory variable, we found no significant differences in the rhizosphere bacterial communities between ZD and ZD91 in the same year (Fig. 5a), but the community structures differed significantly between these 2 years (Fig. 5c). The growth stage also influenced community structures (Fig. 5b). The PCA analysis revealed that the growing year was the major factor that contributes to the difference in community structures. Furthermore, the bacterial community structure in soil was marginally related to the growth stage. We also calculated ADONIS differences between cultivars, growth stages, and growth years, and the interactions among these factors. According to the ADONIS values, the bacterial community structure was strongly influenced by year-to-year variation and growth stage (Table 2).

**Table 2:** ADONIS analysis of effects of cultivar, growth stage, year, and the interactions between them on the bacteria community structure in rhizosphere soil

	F. model	R^2^	Pr (> F)
Growth stage	4.03	0.13	< 0.001
Cultivar	1.09	0.02	0.307
Year	14.64	0.12	< 0.001
Growth stage × cultivar	0.98	0.05	0.524
Growth stage × year	3.00	0.10	< 0.001
Cultivar × year	0.92	0.01	0.487
Growth stage × cultivar × year	1.22	0.03	0.150

### Effects of cultivar, growth stage, and year on the rhizosphere bacterial communities

**Fig. 6:**
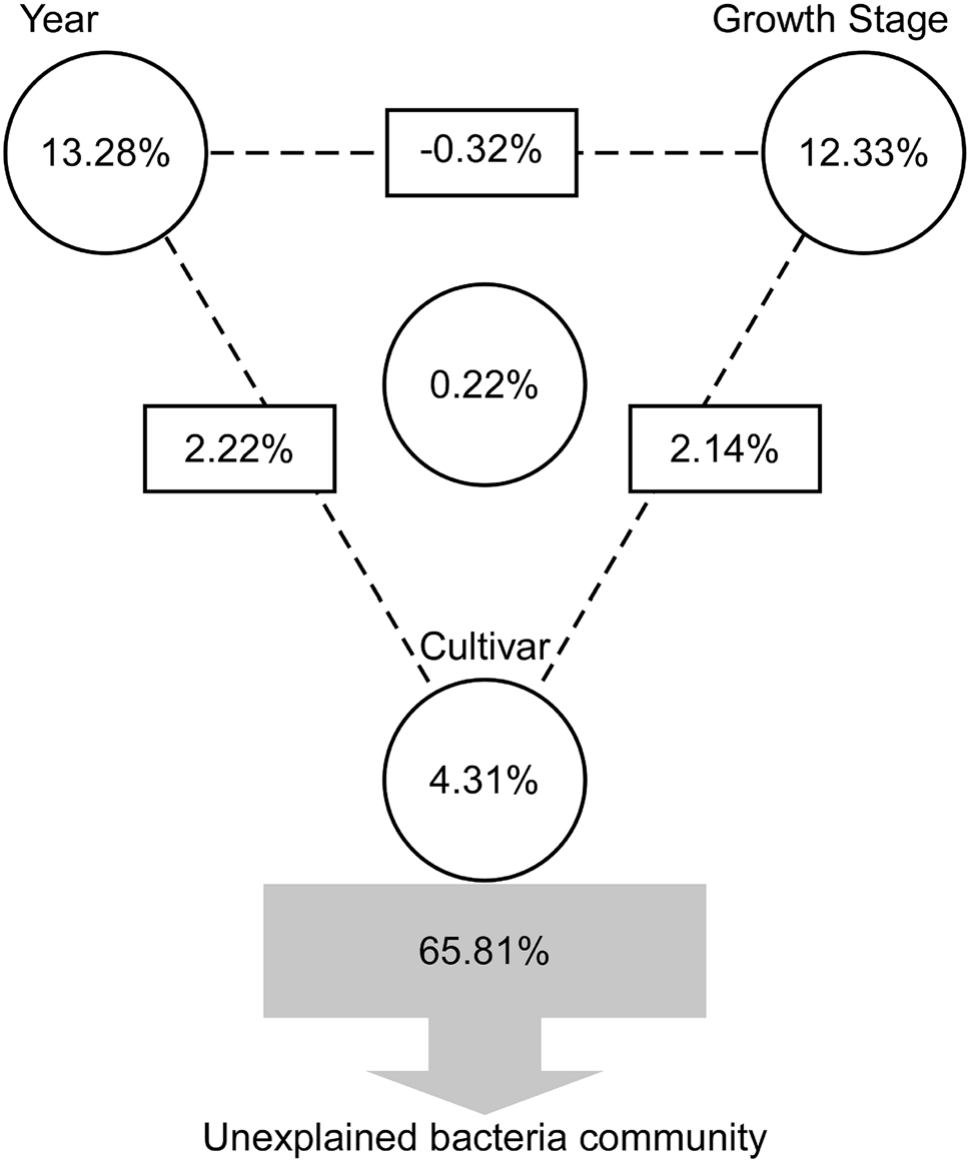
VPA of the bacterial community structure. Effects of cultivar, growth stage, planting year, and the interactions among these factors on the bacterial community structure. Circles on the edges of the triangle show the percentage of variation caused by each factor alone. The percentage of variation caused by interactions between two or three of the factors is shown as squares on the sides and as circles in the center of the triangle. The unexplained variation is depicted in square at the bottom of the figure

To quantify the relative contributions of cultivar genotype, growth stage, and year to the total bacterial community based on OTU composition, we performed the VPA. Variations in the bacterial community structure were partitioned among cultivars, growth stages and years, as well as the interactions among these variables. These variables explained 34.19% of the observed variations, leaving 65.81% of the variations unexplained (Fig. 6). Cultivar explained 4.31% of the variations (*P* = 0.488), and growth stage accounted for 12.33% (*P* = 0.001). Most of the variations (13.28%) occurred in different growing years (*P* = 0.001). The interactions between year and cultivar, and between growth stage and cultivar, accounted for 2.22 and 2.14% of the variations, respectively. Thus, different growth year is the most important factor contributing to the shifts in the bacterial community structure.

## Discussion

The biodiversity of soil microbial communities is an important reference for evaluating the safety of GM crops (Guan et al. 2016; Hannula et al. 2014). In addition, there were large numbers of bacteria flourished in the rhizosphere (Sugiyama et al. 2014). Here, we presented our analysis of the rhizosphere bacterial communities affected by cultivation of the transgenic high-methionine soybean cultivar ZD91 through Illumina MiSeq sequencing of the 16S rRNA gene. This technique provides a sensitive methodology for risk assessment of GM crops (Delgado-Baquerizo et al. 2018; Guan et al. 2016).

It is important to know to what extend microbial communities need to be sampled to accurately measure the changes in diversity. Lundin et al. (2012) found that 1000 denoised sequences per sample explained up to 90% the trends in β-diversity. Similarly, 5000 denoised sequences were sufficient to describe trends in α-diversity. Since an average of 61,255 sequences was obtained per sample in our study, it is sufficed to say that our data is sufficient to describe patterns in the bacterial α- and β-diversity.

For increasing methionine contents in soybean, the cystathionine γ-synthase (AtD-CGS) gene from *Arabidopsis thaliana* was expressed under the control of a seed-specific promoter, legumin B4, in the soybean cultivar ZD (Song et al. 2013). In contrast to certain seed specific promoters that result in exogenous heterotopic expression in other parts of plants, in addition to the seeds (Qu and Takaiwa 2004; Russell and Fromm 1997), this AtD-CGS gene is highly expressed in seeds and the expression is low in other tissues such as stem, root and leaf (Song et al. 2013). Nevertheless, it is suspected that there might be some changes in types and quantities of amino acids in root secretions associated with this transgenic soybean (Huang et al. 2014; Savka and Farrand 1992). Here, we found that there was no differences in methionine contents between ZD and ZD91 in root exudates (Liang et al. 2014b). Accordingly, there was no associated changes found in the bacterial community structure between ZD and ZD91 soybean lines.

In previous studies, we showed that the transgenic soybean ZD91 has no significant impacts on the functional diversity of rhizosphere microorganisms, the arbuscular mycorrhizal fungal community structure, the nitrogen-transforming bacteria, soil organic elements and enzyme activities (Liang et al. 2015, 2016, 2017, 2018). In this study, the sequencing data revealed no significant differences in the rhizosphere structure of the bacterial community between the two cultivars, despite that the structures in the soybean plots differed significantly between the two growing years, and the structure was marginally related to the growth stages (Fig. 5). This is consistent with the previous studies that demonstrate year-to-year variations in soil bacterial communities is not uncommon and plant growth stage influences soil microbiome composition (Cotta et al. 2014; DeBruyn et al. 2017; Hannula et al. 2012; Zhang et al. 2015). Thus, our results demonstrated again that growing years and growth stages rather than plant genotypes (transgenic vs. control) are the main driver for variations in the bacterial community within the rhizosphere.

The taxa Acidobacteria, Proteobacteria, Actinobacteria, Bacteroidetes, Verrucomicrobia, Planctomycetes, and Firmicutes were found to be all prevalent in the rhizosphere. Most of the OTUs existed between year 2012 and year 2013 belonged to Proteobacteria, Planctomycetes, Actinobacteria, Bacteroidetes, and Acidobacteria. The majority of OTUs existed in all four growth stages in year 2012 or year 2013 fall into Proteobacteria, Planctomycetes, Acidobacteria, and Actinobacteria. OTUs occurred in each of the growth stage mostly belong to Proteobacteria.

Similar with our previous findings, these seven phyla constituted the dominant taxonomic groups for ZD and ZD91 in the pod-setting stage (Liang et al. 2014b). Acidobacteria are commonly detected in soils, and members of this phylum are likely to play a role in terrestrial ecosystem processes. Moreover, a previous study showed that members of the Acidobacteria (e.g., subgroups Gp4 and Gp6) on average represent 20% of typical bacterial communities in soil (Babujia et al. 2016). Proteobacteria are critical for soil fertility due to their roles in C, N, and S cycles (Babujia et al. 2016). Actinobacteria is an important component of soil communities (Barriuso et al. 2012). Bacteroidetes are thought to degrade polymers in soil and positively correlated with C mineralization rates. And the active members of this phyla were some of the initial metabolizers of labile carbon inputs (Aislabie et al. 2008; Eilers et al. 2012; Fierer et al. 2007; Lauber et al. 2009). Many soil Verrucomicrobia are oligotrophic and are able to grow under conditions of low C availability (Eilers et al. 2012). In addition, Firmicutes and Planctomycetes were identified as major soil bacterial phyla (Liebner et al. 2008). The overall distribution of these seven phyla did not differ between non-transgenic and transgenic soybean at any sampling time points, but both the plant growth stage and growth year have greater effects on rhizosphere bacterial communities.

Further, a large proportion of the bacterial 16S rRNA gene is unclassified due to, to some degree, low read accuracy and low resolutions of short amplicons (Wagner et al. 2016). This suggests that high throughput full-length bacterial 16S rRNA gene sequencing methodologies with reduced biases are needed. Another possibility is that these unclassified sequences could either have been novel and therefore could not be classified or they belonged to less well-studied lineages (Yang et al. 2015).

In conclusion, our results showed that transgenic soybean ZD91 did not significantly affect the rhizosphere bacterial communities dynamic. The bacterial community structure was markedly affected by natural variations relevant to the planting year and growth stage. This finding contributes to our understanding of what GM crops might impact on the ecosystem and the safety concerns posed by the cultivation of GM crops, which will provide reliable scientific data for consideration of ZD91 commercial cultivation.
